# Supplementary material for: Genome-Wide Mapping Reveals Complex Regulatory Activities of BfmR in Pseudomonas aeruginosa
Source: Microorganisms. 2021 Feb 25;9(3):485. doi: 10.3390/microorganisms9030485 (PMC8025907; doi:10.3390/microorganisms9030485)
Supplement: Supplementary file 1 [file microorganisms-09-00485-s001.zip › microorganisms-1094669-s/microorganisms-1094669-supplementary.docx]

**
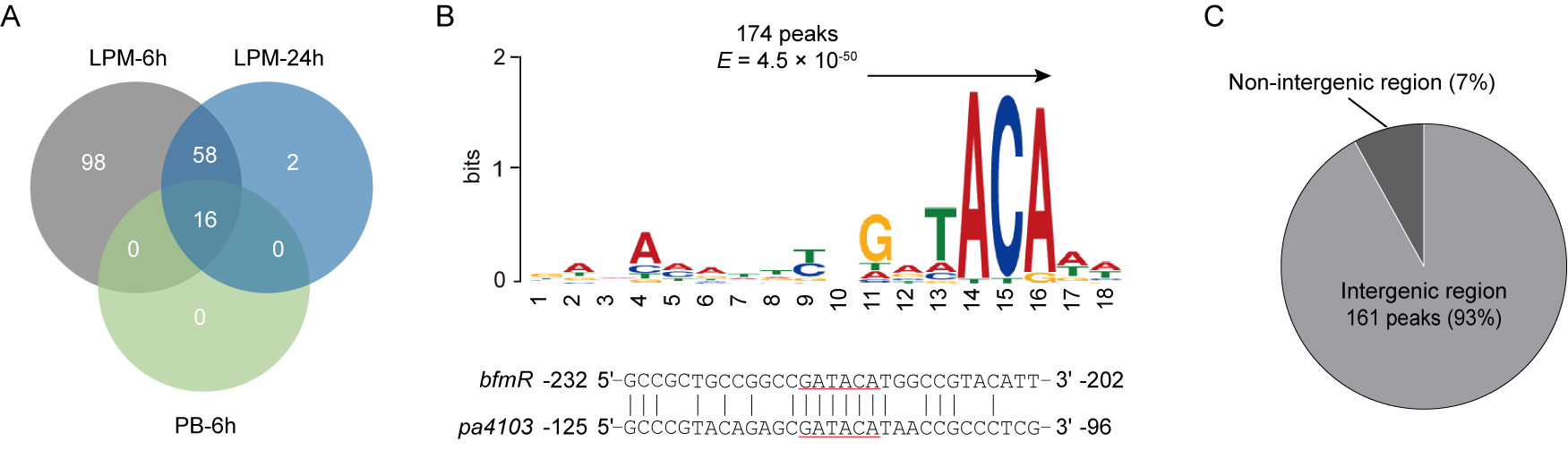
Figure S1. Analysis of BfmR ChIP-seq.** (A) Quantitative analysis of the BfmR binding sites under three different growth conditions. LPM-6h, bacteria were cultured in low-phosphate M8-glutamate minimal medium (LPM) for 6 hours; LPM-24h, bacteria were cultured in LPM for 24 hours; PB-6h, bacteria were cultured in Pyocyanin production broth (PB) for 6 hours. (B) The most significant motif was generated by the MEME tool using 101 bp centered on the peak summit of 174 peak sequences (Data set 2). The height of each letter represents the frequency of each base in different locations in the consensus sequence. The location of the conserved promoter element generated by MEME (*upper panel*) on the BfmR-protected regions of *bfmR* and *pa4103* promoters (1) are underlined (*lower panel*). (C) Pie chart showing BfmR binding sites relatively to the nearest gene. BfmR binding sites refer to the 101 bp fragment centered on the peak summit are shown in in Data set 2. Intergenic region represents that BfmR binding sites are located in promoter of nearest gene (and/or operon) or downstream of the nearest gene. Non-intergenic region represents that BfmR binding site are located inside or overlap end of the nearest gene.

**
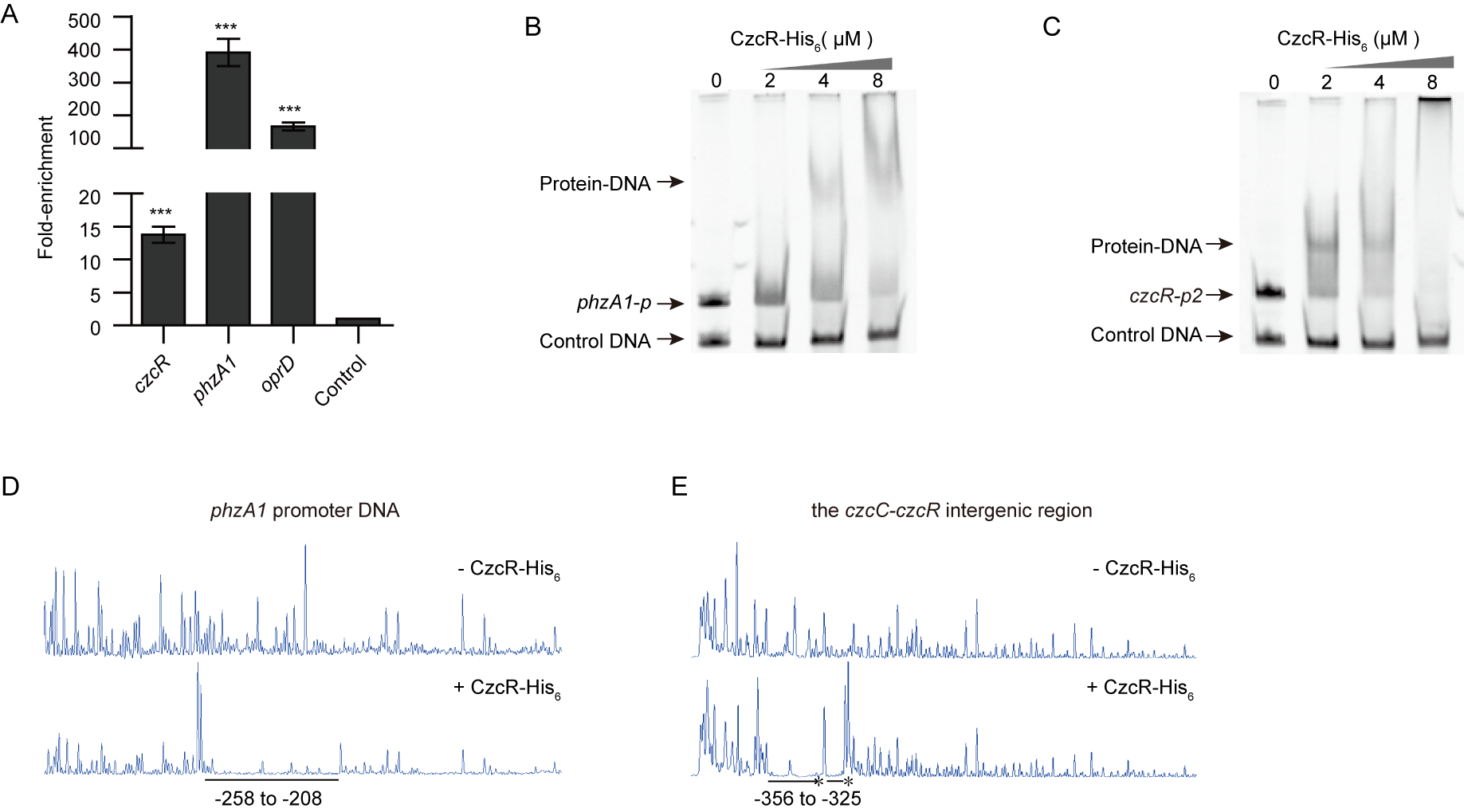
Figure S2. Validation of the binding of CzcR to selected target regions.** (A) ChIP-Quantitative PCR (ChIP-qPCR) analysis of CzcR binding to the promoter of selected genes (i.e., *czcR*, *phzA1*, and *oprD*). C-*phoB* serves as a negative control and the results are reported as fold changes with control (ChIP-DNA vs. Input-DNA) set to 1. ***P < 0.001, Student's two-tailed *t*-test. Results represent means ± SD (n=3 biological replicates). (B and C) EMSA showing the binding of C-terminal His_6_-tagged CzcR (CzcR-His_6_) to the promoter DNAs of *phzA1* (i.e., *phzA1*-*p*) (B) and *czcR* (i.e., *czcR-p2*) (C). DNA fragments C-*phzS* serve as a negative control. (D and E) Electropherograms show the protection pattern of *phzA1* promoter (D) and the *czcC*-*czcR* intergenic region (E) after digestion with DNase I following incubation in the absence (-) and presence (+) of CzcR-His_6_ (18 μM). The protected regions (relative to the start codon of *phzA1* and *czcR*) are underlined, and asterisk indicates DNase I hypersensitivity site.

**
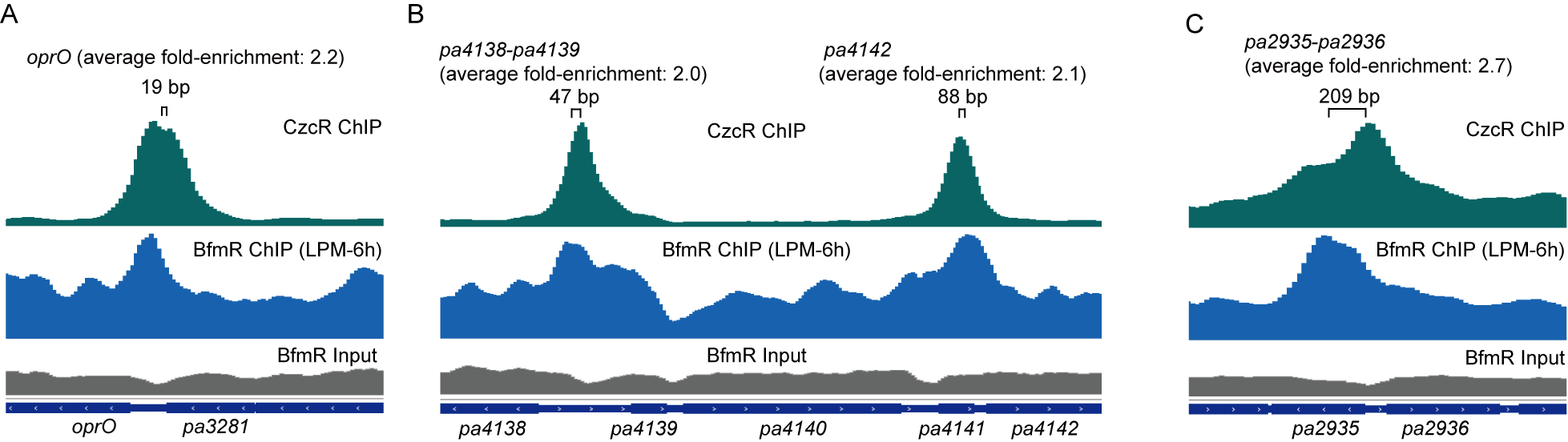
Figure S3. Comparison of BfmR and CzcR binding sites.** Pattern of CzcR (green) and BfmR (blue) binding peaks identified upstream of the *oprO* (A), in the intergenic region between *pa4138* and *pa4139* and the upstream of *pa4142* (B), and in the intergenic region between *pa2935* and *pa2936* (C). The input control sample were shown in gray. The distance between CzcR and BfmR ChIP-seq peak summits are indicated by brackets and numbers. The average fold-enrichment was generated by three replicates of ChIP-seq (LPM-6h).

**
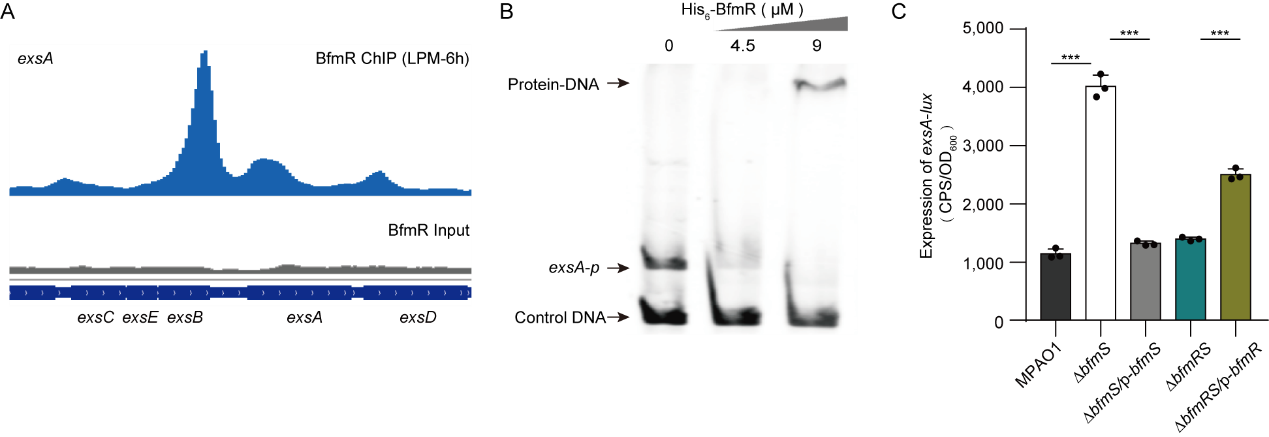
Figure S4. BfmR binds to *exsA* promoter region and controls its activity.** (A) Pattern of BfmR ChIP-seq peaks identified upstream of the *exsA*. ChIP sample (blue) and the input control sample (grey) were shown. (B) An EMSA experiment showed that His_6_-BfmR binds to the promoter region of *exsA* (i.e., *exsA*-*p*). DNA fragment C-*bfmR* serves as a negative control. (C) The promoter activity of *exsA* in WT MPAO1 and its derivatives grown in minimal medium (MM) at 37 °C for 6 h. MPAO1, Δ*bfmS*, and Δ*bfmRS* harboring an empty pAK1900 vector; p-*bfmS* and p-*bfmR* denote pAK1900-*bfmS* and pAK1900-*bfmR* (Table S1), respectively. Data points are shown in black dots, ***P < 0.001, Student's two-tailed *t*-test. Results represent means ± SD (n = 3 biological replicates).

**
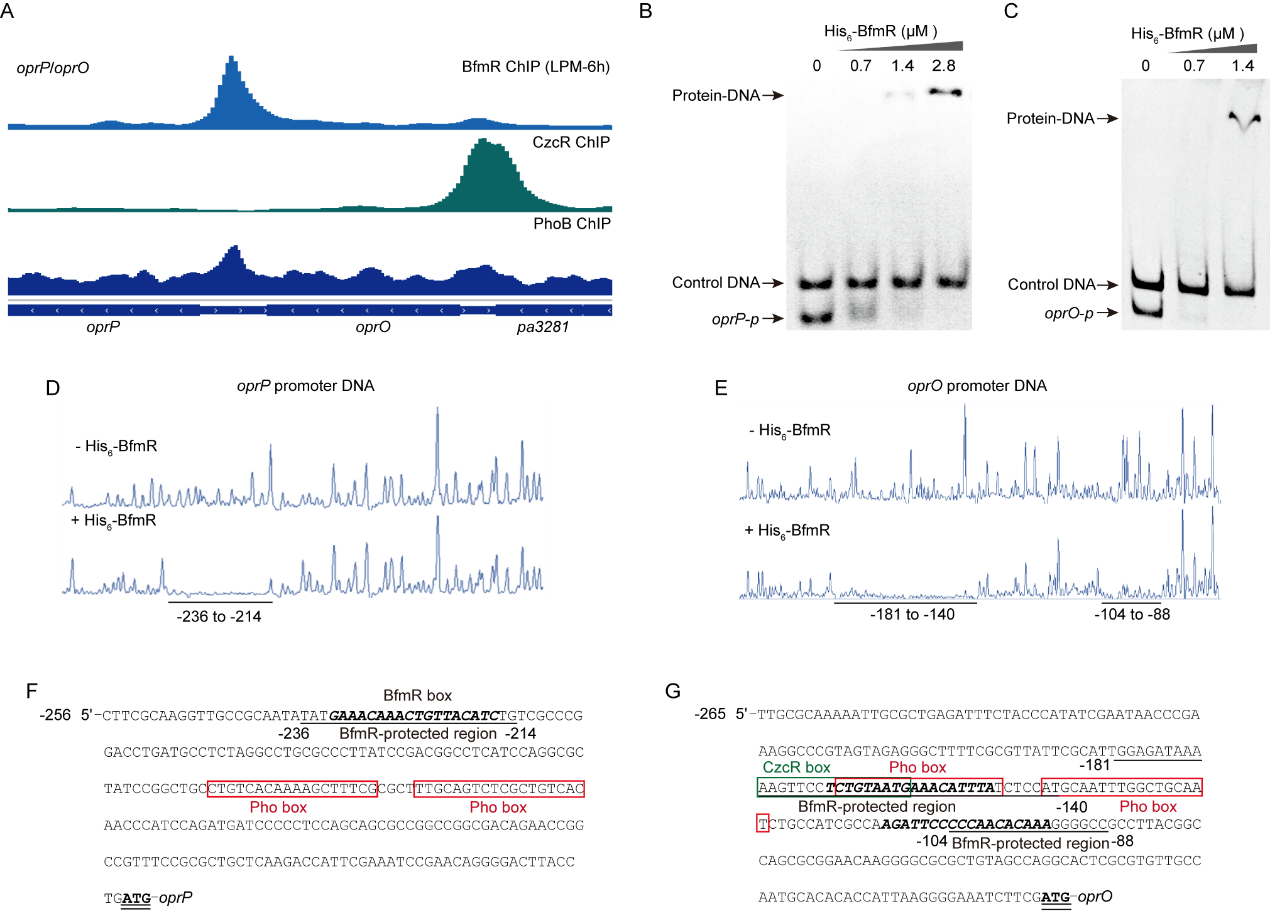
Figure S5.** **BfmR binds to the potential promoter region of *oprP* and *oprO*.** (A) BfmR, CzcR and PhoB ChIP-seq signals upstream of the *oprP* or *oprO*. The PhoB ChIP data were obtained from GEO (accession number GSE128430) (2). (B and C) EMSA experiments showed that His_6_-BfmR binds to the promoter region of *oprP* (*oprP*-*p*) (B) and *oprO* (*oprO*-*p*) (C), DNA fragment C-*rhlC* serves as the negative control. (D and E) Electropherograms show the protection pattern of *oprP*-*p* (D) and *oprO*-*p* (E) after digestion with DNase I following incubation in the absence (-) and presence (+) of His_6_-BfmR (6 μM). The protected regions (relative to the start codon of *oprP* and *oprO*) are underlined. (F and G) *oprP* (F) and *oprO* (G) promoter sequences with a summary of the DNase I footprint assay and ChIP-seq results. The BfmR-protected regions are underlined, as indicated. Sequences that match the MEME motifs of BfmR (Figure 1C) are in bold and italic. The potential Pho box (3) is highlighted by square frame, CzcR box are indicated by green square frame according to the MEME result; the starting codon of *oprP* and *oprO* (ATG) are in bold and double underlined.

**
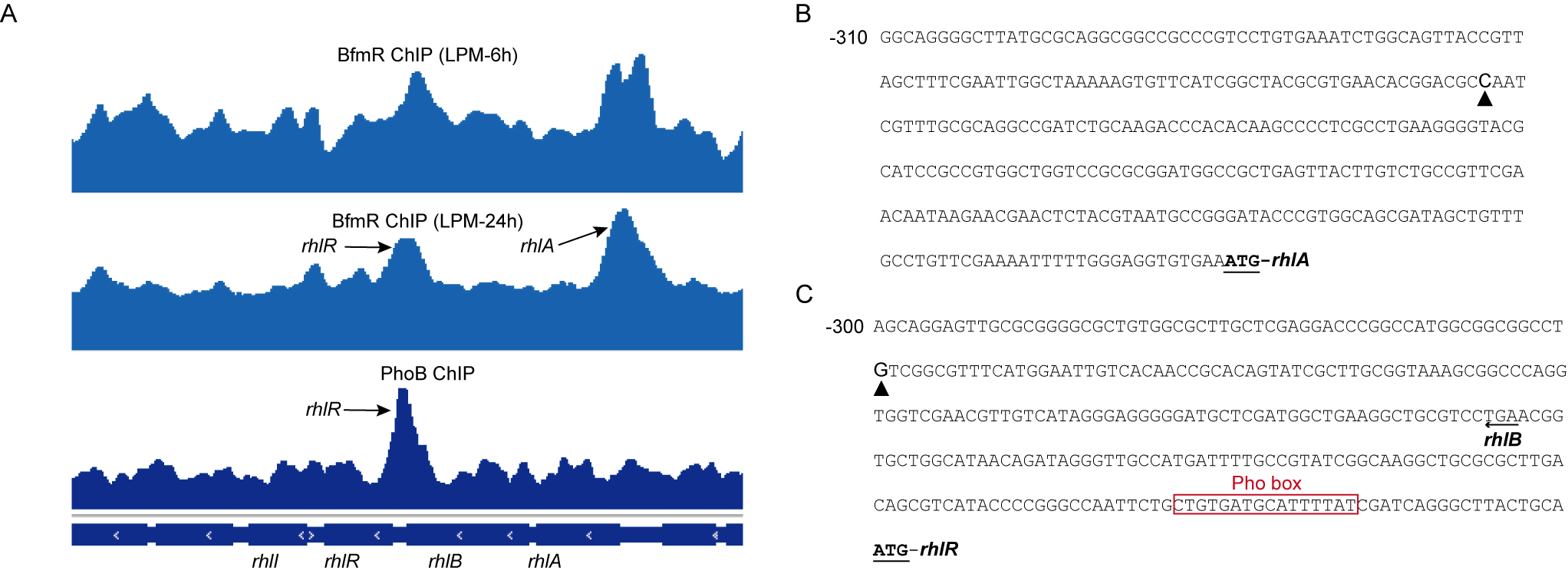
Figure S6. BfmR and PhoB ChIP-seq signals on the promoters of *rhlR* and *rhlA*.** (A) Pattern of BfmR (LPM-6h and LPM-24h) and PhoB ChIP-seq peaks located upstream of *rhlA* and *rhlR*. The PhoB ChIP data were obtained from GEO (accession number GSE128430) (2). (B and C) Sequence up of *rhlA* (B) and *rhlR* (C) with a summary of ChIP-seq results. The triangle indicates BfmR ChIP-seq peak summit, the potential Pho box (3) is highlighted by square frame, and the starting codon (ATG) of *rhlA* and *rhlR* are in bold and underlined.

**
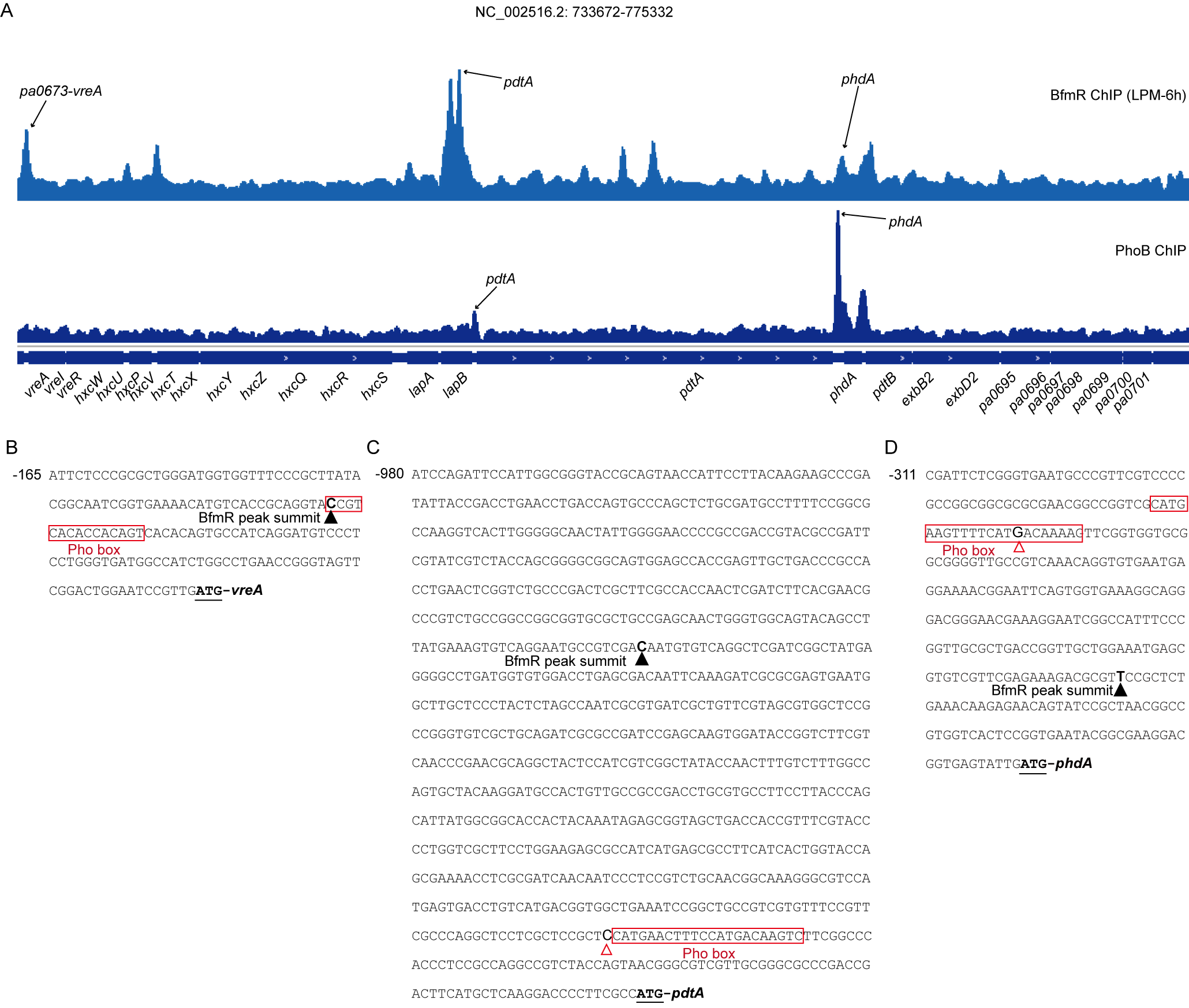
Figure S7. ChIP-seq signals and promoter sequences with a summary of the ChIP-seq results.** (A) BfmR (LPM-6h and LPM-24h) and PhoB ChIP-seq signal on the *vreAIR* locus encoding the PUMA3 cell-surface signalling system and the PUMA3-regulated genes; the arrow indicates the peak position. The PhoB ChIP data were obtained from GEO (accession number GSE128430) (2). (B) Upstream sequence of *vreAIR* operon with a summary of the BfmR ChIP-seq result. (C) Upstream sequence of *pdtA* with a summary of the ChIP-seq results. (D) Upstream sequence of *phdA* with a summary of the ChIP-seq results. In (B), (C), and (D), the BfmR and PhoB ChIP-seq peak summits are indicated by solid and red hollow triangle, respectively, and the potential Pho box (3–5) is highlighted by square frame; the starting codon (ATG) are in bold and underlined.

**
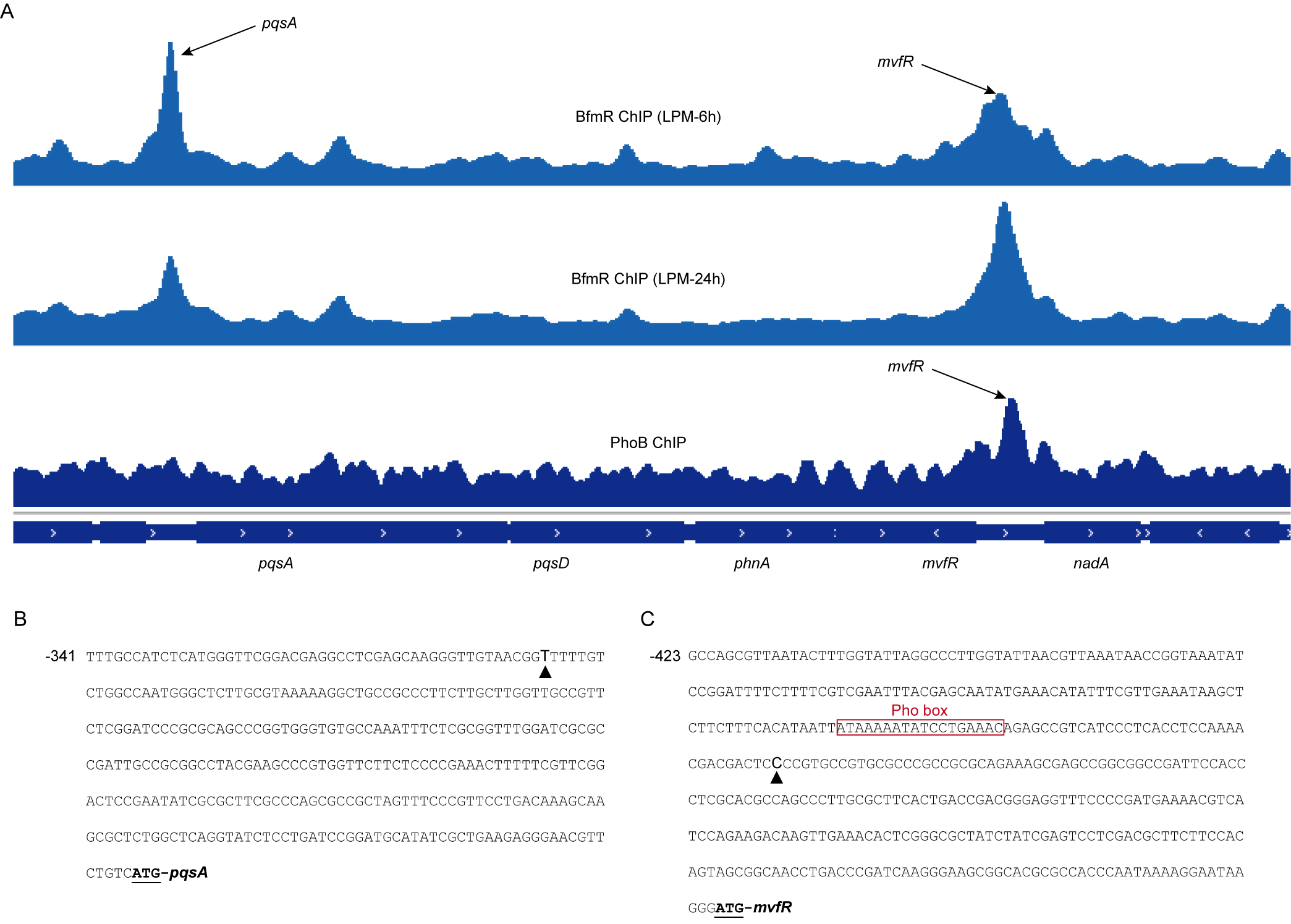
**

**Figure S8. Comparison of BfmR and PhoB binding sites on the promoters of *pqsA* and *mvfR*.** (A) Pattern of BfmR and PhoB ChIP-seq peaks identified upstream of *pqsA* and *mvfR*. The PhoB ChIP data were obtained from GEO (accession number GSE128430) (2). (B and C) The potential promoter region of *pqsA* (B) and *mvfR* (C). BfmR ChIP-seq peak summit on the sequence is indicated by the triangle. The potential Pho box (3) is highlighted by square frame, and the starting codon (ATG) of *pqsA* and *mvfR* are in bold and underlined.

**Table S1. Plasmids and bacterial strains used in this study**

| Plasmids or strains | Relevant characteristics^a^ | Source |
| --- | --- | --- |
| **Plasmids** |  |  |
| pAK1900 | *Escherichia coli*–*Pseudomonas aeruginosa* shuttle cloning vector, Ap^r^ Cb^r^ | (6) |
| pEX18Ap | Gene replacement vector, mob^+^*sacB*, Ap^r^ | (7) |
| pPS858 | pBR322 derivative carrying a FRT-Gm cassette, Ap^r^ | (7) |
| pFLP2 | Source of Flp recombinase; Ap^r^/Cb^r^ | (7) |
| pET22b(+) | Carries a T7 *lac* promoter-operator and an N-terminal *pelB* signal sequence for potential periplasmic localization, plus optional C-terminal 6His Tag sequence, Ap^r^ | Novagen |
| pET28a | T7 *lac* promoter–operator, N-terminal His tag, kan^r^ | Novagen |
| mini-CTX-*lacZ* | Gene delivery vector for inserting genes at the CTX phage *att* site on *P. aeruginosa* chromosome, Tc^r^; *lacZ-*based promoter reporter | (8) |
| mini-CTX-*lux* | Gene delivery vector for inserting genes at the CTX phage *att* site on *P. aeruginosa* chromosome, Tc^r^; *lux-*based promoter reporter | (8) |
| p-*bfmS* | pAK1900 derivative carrying *bfmS* (*pa4102*) on a *~*1.4 kb *Hin*dIII/*Bam*HI fragment in same orientation as p*lac* | (1) |
| p-*bfmR* | pAK1900 derivative carrying *bfmR* (*pa4101*) on a ~0.8 kb *Hin*dIII/*Bam*HI fragment in same orientation as p*lac* | (1) |
| p-*czcS* | pAK1900 derivative carrying *czcS* (pa2523) on a ~1.4 kb HindIII/BamHI fragment in same orientation as *plac* | This study |
| p-*phoB* | pAK1900 derivative carrying *bfmR* (*phoB*) on a *~*0.8 kb *Hin*dIII/*Bam*HI fragment in same orientation as p*lac* | This study |
| *czcC*-*lux* | mini-CTX-*lux* containing carrying *czcC* promoter region(- 825 to + 135 of the start codon) | This study |
| *czcR*-*lux* | mini-CTX-*lux* containing carrying *czcR* promoter region(- 647 to + 313 of the start codon) | This study |
| *exsA*-*lux* | mini-CTX-*lux* containing carrying *exsA* promoter region(-366 to +26 of the start codon) | This study |
| mini-*phoB*-*flag* | mini-CTX-*lacZ* derivative carrying *phoB-flag* (covering the region from 561 bp upstream and the *phoB* gene) | This study |
| mini-*czcR*-*flag* | mini-CTX-*lacZ* derivative carrying *czcR-flag* (covering the region from 512 bp upstream and the *czcR* gene) | This study |
| mini-*bfmR*-*flag* | mini-CTX-*lacZ* derivative carrying *bfmR-flag* | (1) |
| mini-*phoB*-D-*flag* | mini-CTX-*lacZ* derivative carrying *phoB*-*flag* (covering the region from 561 bp upstream and the *phoB* gene) that lacks the putative BfmR-binding site GACACA) | This study |
| 28a-6His-BfmR | pET28a derivative carrying a full-length of *bfmR* for the expression of an N-terminal 6His-tagged of BfmR (His_6_-BfmR) | (1) |
| 22b-CzcR-6His | pET22b (+) derivative carrying a full-length *czcR* for the expression of a C-terminal 6His-tagged CzcR (CzcR-His_6_) | This study |
| pEX18Ap::*bfmRS*UGD | pEX18Ap derivative, for replacing *bfmRS* loci with a gentamicin resistance cassette from plasmid pPS858 | (1) |
| pEX18Ap::*bfmS*UGD | pEX18Ap derivative, for replacing *bfmS* gene with a gentamicin resistance cassette from plasmid pPS858 | (1) |
| pEX18Ap::*phoB*UGD | pEX18Ap derivative, for replacing *phoB* loci with a gentamicin resistance cassette from plasmid pPS858 | This study |
| pEX18Ap::*czcRS*UGD | pEX18Ap derivative, for replacing *czcRS* loci with a gentamicin resistance cassette from plasmid pPS858 | This study |
| ***P. aeruginosa* strains** |  |  |
| MPAO1 | Wild type | (9) |
| Δ*bfmS* | MPAO1 derivative with a gentamicin resistance cassette replaced the *bfmS* gene | (1) |
| Δ*bfmRS* | MPAO1 derivative with a gentamicin resistance cassette replaced the *bfmRS* locus | (1) |
| Δ*czcRS* | *czcRS* deletion mutant of MPAO1 | This study |
| Δ*phoB* | *phoB* deletion mutant of MPAO1 | This study |
| Δ*phoB*Δ*bfmS* | Δ*phoB* derivative with a gentamicin resistance cassette replaced the *bfmS* | This study |
| MPAO1/pAK1900 | MPAO1 carrying plasmid pAK1900 | (1) |
| Δ*bfmS*/pAK1900 | Δ*bfmS* carrying plasmid pAK1900 | (1) |
| Δ*bfmRS*/pAK1900 | Δ*bfmRS* carrying plasmid pAK1900 | (1) |
| Δ*bfmS*/p-*bfmS* | Δ*bfmS* carrying plasmid p-*bfmS* | (1) |
| Δ*bfmRS*/p-*bfmR* | Δ*bfmRS* carrying plasmid p-*bfmR* | (1) |
| Δ*phoB*Δ*bfmS*/pAK1900 | Δ*phoB*Δ*bfmS* carrying plasmid pAK1900 | This study |
| Δ*phoB*Δ*bfmS*/p-*phoB* | Δ*phoB*Δ*bfmS* carrying plasmid p-*phoB* | This study |
| Δ*bfmRS*/mini-*bfmR*-*flag* | Δ*bfmRS* carrying plasmids of mini-*bfmR*-*flag* | (1) |
| Δ*czcRS*/p-*czcS* + mini-*czcR*-*flag* | Δ*czcRS* carrying plasmids of pAK1900::*czcS* and mini-*czcR*-*flag* | This study |
| MPAO1/pAK1900 + *czcC*-*lux* | MPAO1 carrying plasmids of pAK1900 and *czcC*-*lux* | This study |
| Δ*bfmS*/pAK1900 + *czcC*-*lux* | Δ*bfmS* carrying plasmids of pAK1900 and *czcC*-*lux* | This study |
| Δ*bfmRS*/pAK1900 + *czcC*-*lux* | Δ*bfmRS* carrying plasmids of pAK1900 and *czcC*-*lux* | This study |
| Δ*bfmS*/p-*bfmS* + *czcC*-*lux* | Δ*bfmS* carrying plasmids of p-*bfmS* and *czcC*-*lux* | This study |
| Δ*bfmRS*/p-*bfmR* + *czcC*-*lux* | Δ*bfmRS* carrying plasmids of p-*bfmR* and *czcC*-*lux* | This study |
| MPAO1/pAK1900 + *czcR*-*lux* | MPAO1 carrying plasmids of pAK1900 and *czcR*-*lux* | This study |
| Δ*bfmS*/pAK1900 + *czcR*-*lux* | Δ*bfmS* carrying plasmids of pAK1900 and *czcR*-*lux* | This study |
| Δ*bfmRS*/pAK1900 + *czcR*-*lux* | Δ*bfmRS* carrying plasmids of pAK1900 and *czcR*-*lux* | This study |
| Δ*bfmS*/p-*bfmS* + *czcR*-*lux* | Δ*bfmS* carrying plasmids of p-*bfmS* and *czcR*-*lux* | This study |
| Δ*bfmRS*/p-*bfmR* + *czcR*-*lux* | Δ*bfmRS* carrying plasmids of p-*bfmR* and *czcR*-*lux* | This study |
| MPAO1/pAK1900 + mini-*phoB*-*flag* | MPAO1 carrying plasmids of pAK1900 and mini-*phoB*-*flag* | This study |
| Δ*bfmS*/pAK1900 + mini-*phoB*-*flag* | Δ*bfmS* carrying plasmids of pAK1900 and mini-*phoB*-*flag* | This study |
| Δ*bfmRS*/pAK1900 + mini-*phoB*-*flag* | Δ*bfmRS* carrying plasmids of pAK1900 and mini-*phoB*-*flag* | This study |
| Δ*bfmS*/p-*bfmS* + mini-*phoB*-*flag* | Δ*bfmS* carrying plasmids of p-*bfmS* and mini-*phoB*-*flag* | This study |
| MPAO1/pAK1900 + mini-*phoB*-D-*flag* | MPAO1 carrying plasmids of pAK1900 and mini-*phoB*-D-*flag* | This study |
| Δ*bfmS*/pAK1900 + mini-*phoB*-D-*flag* | Δ*bfmS* carrying plasmids of pAK1900 and mini-*phoB*-D-*flag* | This study |
| Δ*bfmRS*/pAK1900 + mini-*phoB*-D-*flag* | Δ*bfmRS* carrying plasmids of pAK1900 and mini-*phoB*-D-*flag* | This study |
| Δ*bfmS*/p-*bfmS* + mini-*phoB*-D-*flag* | Δ*bfmS* carrying plasmids of p-*bfmS* and mini-*phoB*-D-*flag* | This study |
| MPAO1/pAK1900 + *exsA*-*lux* | MPAO1 carrying plasmids of pAK1900 and *exsA*-*lux* | This study |
| Δ*bfmS*/pAK1900 + *exsA*-*lux* | Δ*bfmS* carrying plasmids of pAK1900 and *exsA*-*lux* | This study |
| Δ*bfmRS*/pAK1900 + *exsA*-*lux* | Δ*bfmRS* carrying plasmids of pAK1900 and *exsA*-*lux* | This study |
| Δ*bfmS*/p-*bfmS* + *exsA*-*lux* | Δ*bfmS* carrying plasmids of p-*bfmS* and *exsA*-*lux* | This study |
| Δ*bfmRS*/p-*bfmR* + *exsA*-*lux* | Δ*bfmRS* carrying plasmids of p-*bfmR* and *exsA*-*lux* | This study |
| ***E. coli* strains** |  |  |
| DH5a | *endA hsdR17 supE44 thi-1 recA1 gyrA relA1*Δ(*lacZYA-argF*)*U169 deoR* (*φ80dlac*Δ(*lacZ*)*M15*) | Lab stock |
| BL21 | F^–^*ompT hsdS*_B_ (r_B_^-^ m_B_^-^) *gal dcm met* (DE3) | Lab stock |
| S17 λ-pir | *recA thi pro hsdR*^−^*M*^+^ RP4-2-Tc::Mu Km::Tn*7* λpir (Tp^r^ Str^r^) | Lab stock |

^a^Ap^r^, ampicillin resistance; Cb^r^, carbenicillin resistance; Kan^r^, kanamycin resistance; Tc^r^, tetracycline resistance; Tp^r^, trimethoprim resistance

**Table S2. Primers and fragments used in this study**.

| **Name** | **Oligonucleotide sequence (5' to 3')** |
| --- | --- |
| D-*czcRS*-up-F | 5’-CCGGAATTCAGATGCTGCCGGCCTTGAC-3’ (*Eco*RI) |
| D-*czcRS*-up-R | 5’-CGCGGATCCAGGCCCTGGTGCAGGTAGT-3’ (*Bam*HI) |
| D-*czcRS*-down-F | 5’-CGC GGATCCTACTCGCCGCAGAAACCGGC-3’ (*Bam*HI) |
| D-*czcRS*-down-R | 5’-CCCAAGCTTCGGCGCAATTCAACCTGCCG-3’ (*Hin*dIII) |
| D-*phoB*-up-F | 5’-CCGGAATTCATCCTGATGGCCTTCAC CGC-3’ (*Eco*RI) |
| D-*phoB*-up-R | 5’-CGCGGATCCTGCCAACCATGGTCTTGCCT-3’ (*Bam*HI) |
| D-*phoB*-down-F | 5’-CGCGGATCCACCGGCTATCGTTTCTCCAC-3’ (*Bam*HI) |
| D-*phoB*-down-R | 5’-CCCAAGCTTTGGGCGTCGTTACGGATGCT-3’ (*Hin*dIII) |
| comp-*phoB*-F | 5’-CGCGGATCCGTTGTGTCAC ATACCTGACA-3’ (*Bam*HI) |
| comp-*phoB*-R | 5’-CGGGGTACCTTCACGACGGATTGCATCCT-3’ (*Kpn*I) |
| comp-*czcS*-F | 5’-CCCAAGCTTGAAGTCGCCATCTGCCGCCT-3’ (*Hin*dIII) |
| comp-*czcS*-R | 5’-CGCGGATCCTCAGGCAGCGCCCGTCGAA-3’ (*Bam*HI) |
| mini-*czcR*-*flag*-F | 5’-CCCAAGCTTGTTCCGCTCC TCGTCTGCTG-3’ (*Hin*dIII) |
| mini-*czcR*-*flag*-R | 5’-CGCGGATCCTCACTTATCGTCGTCATCCTTGTAATC TCGGCGCGCTTCCAGGA-3’ (*Bam*HI) |
| mini-*phoB*-*flag*-F | 5’-CCCAAGCTTTCTAGTCCATGTCCGGCT-3’ (*Hin*dIII) |
| mini-*phoB*-*flag*-R | 5’-CGCGGATCCTCACTTATCGTCGTCATCCTTGTAATC GCTCTTGGTGGAG-3’ (*Bam*HI) |
| mini-*phoB*-D-*flag*-F | 5’-CGTTGTGTCACATACCTATTTCGTTATCTAATGC-3’ |
| mini-*phoB*-D-*flag*-R | 5’-GCATTAGATAACGAAATAGGTATGTGACACAACG-3’ |
| mini-*czcC*-*lux*-F | 5’-CCCAAGCTTGGAACTGGAACGGCTTGACC-3’ (*Hin*dIII) |
| mini-*czcC*-*lux*-R | 5’-CGCGGATCCCTGCTCGAGGCTGATCGAAG-3’ (*Bam*HI) |
| mini-*czcR*-*lux*-F | 5’-CCCAAGCTTCTGCTCGAGGCTGATCGAAG-3’ (*Hin*dIII) |
| mini-*czcR*-*lux*-R | 5’-CGCGGATCCGGAACTGGAACGGCTTGACC-3’ (*Bam*HI) |
| mini-*exsA*-*lux*-F | 5’-CCCAAGCTTCTGGTTCGGCGCGACTGTTT-3’ (*Hin*dIII) |
| mini-*exsA*-*lux*-R | 5’-CGCGGATCCCGGCCAAGAGATTTGGCTCC-3’ (*Bam*HI) |
| 22b-*czcR*-F | 5’-CGCCATATGATGCGCATCCTTATTATCG-3’ (*Nde*I) |
| 22b-*czcR*-R | 5’-CCGCTCGAGTCGGCGCGCTTCCAGGA-3’ (*xho*I) |
| EMSA-*czcR* (BfmR)-F | 5’-GATATCTCGCCGGCGATA-3’ |
| EMSA-*czcR* (BfmR)-R | 5’-GTTACATTTCGGGCGTTGCC-3’ |
| EMSA-*czcR* (CzcR)-F | 5’-GGCAGATGTTAGGTAGCGGG-3’ |
| EMSA-*czcR* (CzcR)-R | 5’-GATGCGCATGTTCGCCCCTA-3’ |
| EMSA-*phzA1*-F | 5’-GAGCCTTTTCCTGCGTACCG-3’ |
| EMSA-*phzA1*-R | 5’-TTCGTCGCTCGGTAAGGGAG-3’ |
| EMSA-*oprP*-F | 5’-GAATATCCGAAAGCCAGACG-3’ |
| EMSA-*oprP*-R | 5’-TCTTGAGCAGCGCGGAAAC-3’ |
| EMSA-*oprO*-F | 5’-AAATCCTGCGCGAATACGGCA-3’ |
| EMSA-*oprO*-R | 5’-CGAAGATTTCCCCTTAATGG-3’ |
| EMSA-*bfmR*-F | 5’-CATCGGCCTGGAGATGG-3’ |
| EMSA-*bfmR*-R | 5’-TGAGTACCCGTTGCGGAT-3’ |
| EMSA-*phzS*-F | 5’-GGGCTACTGCCTGTTCGAAC-3’ |
| EMSA-*phzS*-R | 5’-ATGAAACGTCGGCGCAGC-3’ |
| EMSA-*rhlC*-F | 5’-GCTTGCCCTGGTATTCCTC-3’ |
| EMSA-*rhlC*-R | 5’-TCTCCCGTAGGTCGAAGTTG-3’ |
| EMSA-*exsA*-F | 5’-CTGGTTCGGCGCGACTGTTT-3’ |
| EMSA-*exsA*-R | 5’-CGGCCAAGAGATTTGGCTCC-3’ |
| *czcR*-FT (BfmR)-F (FAM) | 5’-GATATCTCGCCGGCGATA-3’ |
| *czcR*-FT (BfmR)-R | 5’-GTTACATTTCGGGCGTTGCC-3’ |
| *czcR*-FT (CzcR)-F (FAM) | 5’-GGCAGATGTTAGGTAGCGGG-3’ |
| *czcR*-FT (CzcR)-R | 5’-GATGCGCATGTTCGCCCCTA-3’ |
| *phoB*-FT-F | 5’-AGCGCCAGGTGGATTCTGTT-3’ |
| *phoB*-FT-R (FAM) | 5’-TTGCGGTCGACGATCAC-3’ |
| *phzA1*-FT-F (FAM) | 5’-GAGCCTTTTCCTGCGTACCG-3’ |
| *phzA1*-FT-R | 5’-TTCGTCGCTCGGTAAGGGAG-3’ |
| *oprP*-FT-F | 5’-GAATATCCGAAAGCCAGACG-3’ |
| *oprP*-FT-R (FAM) | 5’-TCTTGAGCAGCGCGGAAAC-3’ |
| *oprO*-FT-F | 5’-AAATCCTGCGCGAATACGGCA-3’ |
| *oprO*-FT-R (FAM) | 5’-CGAAGATTTCCCCTTAATGG-3’ |
| RT-*czcR*-F | 5’-TGCGGCAGATGTTAGGTAGC-3’ |
| RT-*czcR*-R | 5’-GCCGAAGTGTAACCGCTA-3’ |
| RT-*phzA1*-F | 5’-CACTTCACGGAATGACGCTG-3’ |
| RT-*phzA1*-R | 5’-GGAGTTTCATCCCGGGTTTC-3’ |
| RT-*oprD*-F | 5’-GCACGGAGTTTGCTTATACC-3’ |
| RT-*oprD*-R | 5’-GTTGAGGTCGCTTCTCAAGT-3’ |
| RT-*phoB*-F | 5’-ACGAAGCACCGATTCGCGAG-3’ |
| RT-*phoB*-R | 5’-CGTGCGCCTGCTGGGTATTT-3’ |
| *czcR*-*p1* | a 372-bp DNA fragment covering the promoter region of *czcR* (from -434 to -63 of the start codon) |
| *czcR*-*p2* | a 401-bp DNA fragment covering the promoter region of *czcR* (from -392 to +9 of the start codon) |
| *phzA1*-*p* | a 410-bp DNA fragment covering the promoter region of *phzA1* (from -491 to -82 of the start codon) |
| *exsA*-*p* | a 392-bp DNA fragment covering the promoter region of *exsA* (from -366 to +26 of the start codon) |
| *oprP*-*p* | a 287-bp DNA fragment covering the promoter region of *oprP* (from -318 to -32 of the start codon) |
| *oprO*-*p* | a 421-bp DNA fragment covering the promoter region of *oprO* (from -421 to -1 of the start codon) |
| C-*bfmR* | a 272-bp DNA fragment covering the coding region of *bfmR* (from +279 to +550 of the start codon) |
| C-*phzS* | a 299-bp DNA fragment covering the promoter region of *phzS* (from -367 to -69 of the start codon) |
| C-*rhlC* | a 540-bp DNA fragment covering the promoter region of *rhlC* (from -549 to -10 of the start codon) |

**References**

1. Q. Cao *et al.*, A novel signal transduction pathway that modulates rhl quorum sensing and bacterial virulence in Pseudomonas aeruginosa. *PLoS Pathog* **10**, e1004340 (2014).

2. H. Huang *et al.*, An integrated genomic regulatory network of virulence-related transcriptional factors in Pseudomonas aeruginosa. *Nat Commun* **10**, 2931 (2019).

3. V. Jensen *et al.*, RhlR expression in Pseudomonas aeruginosa is modulated by the Pseudomonas quinolone signal via PhoB-dependent and -independent pathways. *J Bacteriol* **188**, 8601-8606 (2006).

4. L. M. Faure, M. A. Llamas, K. C. Bastiaansen, S. de Bentzmann, S. Bigot, Phosphate starvation relayed by PhoB activates the expression of the Pseudomonas aeruginosa sigmavreI ECF factor and its target genes. *Microbiology* **159**, 1315-1327 (2013).

5. J. M. Quesada, J. R. Otero-Asman, K. C. Bastiaansen, C. Civantos, M. A. Llamas, The Activity of the Pseudomonas aeruginosa Virulence Regulator σ(VreI) Is Modulated by the Anti-σ Factor VreR and the Transcription Factor PhoB. *Front Microbiol* **7**, 1159 (2016).

6. I. Jansons *et al.*, Deletion and transposon mutagenesis and sequence analysis of the pRO1600 OriR region found in the broad-host-range plasmids of the pQF series. *Plasmid* **31**, 265-274 (1994).

7. T. T. Hoang, R. R. Karkhoff-Schweizer, A. J. Kutchma, H. P. Schweizer, A broad-host-range Flp-FRT recombination system for site-specific excision of chromosomally-located DNA sequences: application for isolation of unmarked Pseudomonas aeruginosa mutants. *Gene* **212**, 77-86 (1998).

8. A. Becher, H. P. Schweizer, Integration-proficient Pseudomonas aeruginosa vectors for isolation of single-copy chromosomal lacZ and lux gene fusions. *Biotechniques* **29**, 948-950, 952 (2000).

9. M. A. Jacobs *et al.*, Comprehensive transposon mutant library of Pseudomonas aeruginosa. *Proc Natl Acad Sci U S A* **100**, 14339-14344 (2003).
